# Help-Seeking in Suicidal Situations: Paramount and yet Challenging. Interactions between Significant Others of Suicidal Persons and Health Care Providers

**DOI:** 10.3390/jcm6020017

**Published:** 2017-02-13

**Authors:** Dolores Angela Castelli Dransart, Sophie Guerry

**Affiliations:** HES-SO, University of Applied Sciences and Arts Western Switzerland, School of Social Work Fribourg, 1762 Givisiez, Switzerland; sophie.guerry@hefr.ch

**Keywords:** help-seeking, significant others, suicidal person, cooperation, care providers, suicide, qualitative, communication, informal carers

## Abstract

Significant others are often crucial for suicidal persons or suicide attempters’ access to care, yet little is known about their efforts to seek help. This article presents the findings of a qualitative pilot study carried out in Switzerland on the help-seeking process of 18 significant others, their perception of the care received by their loved one, and the interactions and collaboration they experienced with professionals. Most significant others repeatedly sought out support for their loved one and themselves. The help-seeking process seemed mostly difficult, was seldom successful on the first attempt, and was filled with multiple difficulties, such as availability and continuity of care and cooperation issues with professionals. Two-thirds of participants were not satisfied with the care provided to their loved ones and half of them faced challenges in their cooperation with professionals, i.e., poor sharing of information or not being acknowledged as partners or supported by professionals. Based on their experience, providing education about suicidal crises and care programs to significant others might lighten their burden and improve their cooperation with professionals, who in turn may benefit from training in communication issues and specific methods of cooperation with significant others in suicidal situations.

## 1. Introduction

In its 2014 Report “Preventing suicide: A global imperative” [1], the World Health Organization (WHO) argues that suicide is a global public health issue and that families should be included in suicide prevention and care when appropriate. Indeed, family members are in many cases aware of the suicidal thoughts or behaviours of their loved one [2]. They are often the first ones to whom a suicidal person (i.e., someone who experiences suicidal ideation or suicidal behaviour such as seeking any given suicidal means) or a suicide attempter (i.e., someone who acted out the suicidal ideation and actively tried to put an end to his/her life) [3] will disclose their distress [4] and may witness the suicidal behaviour. A previous study found that family members were present during the suicide attempt in one-third of cases [5].

According to scientific literature, being a relative or friend to a suicidal person or suicide attempter is a particularly difficult and distressing experience, which impacts on emotional and physical health [6,7,8,9,10]. Indeed, this situation requires considerable emotional and practical involvement from relatives and friends and is, in most cases, experienced as a burden [9,10,11], especially when a suicidal person or suicide attempter’s social network is scarce [12] and support or cooperation with professionals is insufficient [9,13]. As mentioned earlier, relatives are often the first ones to pick up the emerging signs of suicidal behaviours or suicidal crises, which is why, in most cases, it is they who first take action and seek professional help [10,11,14,15], in particular because most suicidal persons or suicide attempters are reluctant to do so themselves [16,17,18,19]. Therefore, family and friends are crucial in ensuring that a suicidal person or suicide attempter may access care not only by seeking help themselves but also by encouraging or persuading the suicidal person or suicide attempter to seek or accept help [17].

Despite the very important role family members and friends play in the help-seeking process, little is known about their experiences, needs, and interactions with professionals in this regard [9]. Although scarce, previous research suggests that the help-seeking process is complicated and influenced by an array of factors related to both individual characteristics and preferences of distressed people and their family members and structural aspects [17]. Relatives may not find timely and appropriate support for their loved ones and/or themselves [13]. Moreover, they may become weary or even burnt out if their efforts are not supported by professionals with the consequence that they may give up taking care of or seeking help for the suicidal person. This could increase the risk of suicide for the suicidal person or suicide attempter.

These issues are of particular interest in a country like Switzerland, which had no national suicide prevention strategy until November 2016 and has only a few agencies specifically intended to respond to suicidal behaviours and suicidal persons. Therefore, we decided to conduct a qualitative pilot study on how taking care or supporting a suicidal person or suicide attempter impacted on the life of informal carers and on how they sought help. This article describes the process these people underwent in their attempt to find help for their loved ones; the type of actions taken, the institutions or persons contacted, and the outcomes. It also addresses the issue of families’ and friends’ perception of the care received by their loved one while she/he was suicidal and the interactions and collaboration they experienced with care providers.

## 2. Methods

The study focuses on significant others of suicidal persons or suicide attempters. This comprises family members, partners, friends, or any other person who experiences emotional closeness to the person at risk. In the literature, no distinction is made with regard to various groups of significant others. Therefore, we investigated a variety of people faced with the suicidality of a loved one and adopted a common denomination.

The study aimed at grasping how significant others perceived, were involved in, and dealt with the suicidality (suicidal ideation, suicidal intent and planning, suicide attempt) of loved ones, and what they did (or not) to seek help for their loved one or for themselves.

Significant others were considered as experiential experts, and a qualitative approach allowing a nuanced understanding of the experience lived by significant others (feelings, difficulties, motivations, and actions) “from within” was adopted, given the limited amount of research literature and data on the subject [20].

Participants in the study were significant others of adult (over 18 years) suicidal persons or suicide attempters who had been confronted with suicidal ideation or suicidal behaviour within the five years preceding the study and were living in two French-speaking cantons (states) of Switzerland (Fribourg and Valais). In order for them to be considered as significant others and included in the sample, respondents had to meet two criteria; subjective affective closeness to the suicidal person or suicide attempter (still alive at the time of the interview) on the one hand (feeling emotionally close, caring about the person, having regular contacts with him/her, being concerned about his/her well-being), and having been involved in or having witnessed help-seeking actions for the suicidal person or suicide attempter on the other hand (having supported them, having sought help for them, or having supported/contact with people who supported them).

Participants were recruited through several professional institutions or associations dealing with mental health issues (7), advertisements published in two daily newspapers (10), and posters put up in public university billboards (1). One national association in the social field, two associations in the mental health field (one of them managed by both professionals and informal carers, the other by family members of people with mental health issues), and one association in suicide prevention (managed both by professionals and informal carers), as well as two public mental health agencies and one home care agency funded by the state agreed to help with the recruitment process. Each association or agency designated an intermediary contact person who was specially trained by the authors to identify potential participants meeting the inclusion criteria and to invite them to participate in the study. If the persons agreed to be interviewed, the intermediary person forwarded their contact details to the research team, which then got in touch with them. The contact persons faced a number of refusals. Significant others argued that they were too busy taking care of the suicidal person or suicide attempter to participate or feared exposure. Four people accepted at first and then canceled the interview. Some interviews with participants were also postponed on several occasions. Since only seven participants were recruited in six months through our field partners, advertisements were published in the main newspaper of each state twice (June and September 2007). The recruitment and data collection lasted twelve months, from February 2007 through January 2008.

Twenty-one interviews were conducted, three of them were finally not retained for analysis after finding out that the significant others only had occasional contacts with the suicidal person or suicide attempter (less than once per month) or had been faced with their significant other’s suicidality more than five years before the interview.

Data was collected during semi-structured interviews. This technique allows participants to express themselves freely within a large thematic frame suggested by the interviewer. The interviews lasted 1.5 to 2.5 h and were conducted using an interview guide informed by previous literature on the subject [12,21,22] and completed by the authors. The interview guide contained several sections with open-ended questions relating to the suicidal person or suicide attempter’s situation and suicidality; the support given by the significant others to the suicidal person or suicide attempter as well as the steps taken to look for help; the type of support sought or received by the interviewee and his/her loved one; interactions with professionals; the consequences of the situation for the interviewee (psychological or social impact, consequences on his/her family, etc.); and the different needs felt but not fulfilled. Probes were used to gain deeper insight and understanding of the participants’ narrative or to allow the collection of more precise factual data (concerning, for example, the suicidal scenario or the characteristics of the people whom help was sought out). At the end of the interview, a questionnaire on the socio-demographic data relating to the interviewee and the suicidal person or suicide attempter was filled out with the significant others.

The interviews were conducted at a location chosen by the interviewee, either at the participant’s home (8), at a university office (8), or in other places (2). They were audio-taped, transcribed verbatim and analysed using the procedures of qualitative content analysis [20]. Data was first read repeatedly in order to get a sense of the whole narrative and to build appropriate units of analysis. Descriptive codes were then derived from data by highlighting themes in the verbatim. Descriptive and analytical categories linking or relating to various codes were then sorted out. Analytical syntheses were produced by reorganising the categories of codes and building a typology, which conceptually linked these categories and allowed modeling [23,24,25]. Analysis was performed first within each interview by coding themes and following their unfolding along the narrative in order to grasp the logics and their contextualised meaning. A synthetic document was written for each interview according to the themes, codes, and categories. A cross-sectional analysis of the synthesis was then performed in order to identify analytical codes, which were subsequently reorganised into core-categories and typologies by using a mind mapping software [26]. The code scheme was mainly inductive and created during data analysis, although large categories derived from literature (such as burden, impact of the suicidality on significant others, and access to care) were also subsequently integrated when present in the data. This is considered to be a mixed approach by Miles and Huberman [25]. Triangulation between the authors was performed to reach an agreement with regard to the resulting themes and categories.

The research work was conducted with special consideration to the preservation of dignity, with the free and informed consent of the participants (consent form signed), while wholly abiding by the rules of confidentiality, anonymity, and data protection according to the Fribourg State Law. Ethical approval was given to the research protocol by the Ethics Committees of the cantons of Fribourg and Valais. The contact details of an independent psychologist and psychotherapist (external to the study) were communicated to the interviewees at the end of the interview for a free support session if required. No participant took up this offer. Emotional support was also provided when needed by the authors, a social worker (DACD), and a psychologist (SG).

## 3. Results

The results presented here come from eighteen interviews conducted in French with sixteen women and two men and concerned nineteen situations (one person having discussed two situations involving a suicidal person within her family). The mean age of the significant others was 44 (range 23–61, born between 1946 and 1984). Out of the eighteen interviewees, three had been diagnosed with mental health disorders themselves. At the time of study, participants responded either as spouses or partners (5), children (3), mothers (3), sisters (3), ex-spouses (2 were still married to the suicidal person or suicide attempter at the time of suicidal ideation or behavior but were not living with the suicidal person or suicide attempter any more), or friends (2) of the suicidal person or suicide attempter. The latter were included in the sample because they felt very close to the suicidal person or suicide attempter and were involved in the situation daily when the suicidal person or suicide attempter was suicidal and during the time it took for him/her to get better and stop thinking about putting an end to his/her life. They also served the purposes of theoretical sampling according to Strauss and Corbin [27], which aims at contrasting sampling on the basis of concepts relevant to the evolving theory. In our sample, when we compared the help-seeking strategies reported by significant others (type and number of actions undertaken, number of people or professionals sought out) we observed no major differences between significant others of various status (mother, spouse, friends, etc.). At the time of interview, four respondents were single and living alone, eight lived with their spouse, two lived with their children, three lived with a partner and children, one person roomated. Twelve participants lived in the canton of Fribourg, five in the canton of Valais, and one in the cantons of Vaud and Fribourg. Respondents had been in contact with the suicidal person or suicide attempter for 5 to 44 years.

Regarding the frequency of contacts (either in person or by phone), eleven interviewees had daily contacts and seven had frequent contacts (i.e., several times a week) with the suicidal person or suicide attempter. Significant others with less frequent contacts with the suicidal person or suicide attempter reported the same kind of reactions as those with daily contacts (anxiety, sleeping disorders, loss of appetite, limited leisure activities, and being constantly on guard). On some occasions, significant others restricted their contacts with the suicidal person or suicide attempter as a way to limit the emotional and physical impacts on their life. However, not living in the same household was sometimes considered as even more stressful, as it did not allow checking on or providing timely protection to the suicidal person or suicide attempter, causing more anxiety and sometimes intensifying the search for help.

According to the Swiss Federal Statistical Office’s classification [28], thirteen respondents belonged to middle class categories of income, four were young adults still in education (university level), and one was a farmer (lower class income). With regard to the focus of this article, it is important to note that in Switzerland anyone benefits from basic health insurance regardless of socio-economic status; people with low or no income are allowed state financial support to receive such cover, which includes health and mental health care in public facilities.

The suicidal persons or suicide attempters (19 situations) consisted of eleven men and eight women, aged between 19 and 77 at the time of the interview, of which four had never attempted to complete suicide according to their significant other, five had made one attempt, and ten had made several attempts. In eight situations, the suicidal person or suicide attempter was considered by his/her significant other as no longer being suicidal at the time of the interview. A psychiatric disorder had officially been diagnosed in ten out of these nineteen persons. Significant others reported indications of mental health problems in seven out of the nine persons with no formal diagnosis. In eight situations, the persons were receiving disability benefits, among which six cases were for a psychiatric disorder.

In the next sub-sections, we present the significant others’ help seeking process to support the suicidal person or suicide attempter (actions performed, kind of people sought out, and difficulties faced), the significant others’ perception of patient/client care provided to the suicidal person or suicide attempter (access to care, perceived quality of care, and difficulties faced), and the significant others’ perception of their collaboration with professionals (sharing of information, communication, and support from caregivers).

### 3.1. Help-Seeking Process to Support the Suicidal Person/Suicide Attempter

The large majority (15/18) of interviewees declared having spent a considerable amount of time and energy not only providing moral support to the suicidal person or suicide attempter or preventing the acting out of his/her suicidal ideation, but also trying to get help from a third party. Only a minority of interviewees did not actively seek help, and this was due to the fact that the suicidal person or suicide attempter himself/herself or other relatives had done so, or else that intervention had taken place automatically (by professionals during a hospitalisation for example). Different and various sources of help had been sought including people likely to help, techniques/methods aimed at easing the suffering (classical or alternative therapies), or different types of information to help relatives provide better support to the suicidal person or suicide attempter. Psychiatrists and general practitioners (GPs) were the professionals most frequently called upon for intervention. GPs were mainly contacted by those significant others with no previous experience of mental health care providers. Mental health service providers were mostly called upon for people with mental health disorders, since significant others in these situations were more familiar with such services and, in particular, could better identify and decode suicidal signs. Three significant others also contacted psychiatric hospitals or general hospitals directly. Among other professionals/structures less frequently approached were psychosocial services, the police, the legal guardian of the suicidal person or suicide attempter and pharmacists.

Non-professional persons or organisations, such as friends and volunteer associations for suicide prevention were rarely called upon. The actions taken by significant others were mostly phone calls to professionals, health agencies, psychosocial services, the police, or any other professional likely to help. Such phone calls had different aims: to obtain information, to request intervention (police, hospitalisation), to make an appointment for the suicidal person or suicide attempter, etc. About one-quarter of the significant others took the suicidal person or suicide attempter to the hospital themselves. The same percentage sought information on the Web, from professionals, from acquaintances, or in books.

Help-seeking strategies aimed at calling upon professionals were often unsuccessful at first. Several attempts were necessary for the suicidal person or suicide attempter to receive adequate and specific help. For a number of reasons, nearly half of the significant others who sought help were unsuccessful on one or several occasions. Sometimes, significant others did not look for help in the right places or had been inadequately informed. The lack of availability (waiting list) of professionals was another obstacle reported by significant others.

AUM: “*On the day following his suicide attempt, I told myself ‘I really have to find a psychologist or someone’, well, I tried calling some and I was told everywhere ‘there is a 6-month waiting list’*.”

Significant others often felt that help, when found, was inadequate. They wondered whether professionals grasped the severity of the situation.

EAR: “*The real disappointment for me was when her suicide attempt led her to the hospital, but after three days, they just released her and that was it. Yet I told them ‘but listen, she is not ready to get out, we’ve been dealing with this for ten years, you can be sure that she will try again’.*”

Among the obstacles hindering the help-seeking process, one of the major issues was the refusal by the adult suicidal person or suicide attempter to receive professional care. Half of the significant others had been confronted with this issue on a regular basis or at some point in the process. In these situations, communication with professionals proved challenging.

LEY: “*The doctor told me ‘your husband is a grown-up man’, and then that it wasn’t my role to intervene, and then, that they don’t have to take into account what the family has to say.*”

While fully aware that the rights and wishes of the concerned person were to be respected, significant others considered that the suicidality had to be managed in a specific way by professionals, i.e., giving priority to the severity of the suicidal behaviour over free will and taking the experiences of the significant others into account. In fact, they often possessed facts and information not shared by the suicidal person or suicide attempter with professionals (such as previous suicide attempts or suicide planning).

Our results seem to show that the help-seeking road travelled by the significant others of a suicidal person or suicide attempter is, in most cases, tortuous and difficult. This caused numerous emotional reactions in significant others: they often felt helpless, powerless, disillusioned, desperate, angry; they felt that they were, in practice, not listened to, understood, or helped by the professionals or institutions that they had contacted.

OAM: “A feeling of not being listened to, of not knowing where to go, whom to reach out to, how to find help...we are alone, powerless, we don’t know what to do.”

Confronted with these multiple obstacles and difficulties while trying to find help, almost two-thirds of the interviewees felt gradually weary and exhausted. Some ceased to seek help for the suicidal person or suicide attempter.

ION: “*I tried very hard to find help during this time and after a while, because we can’t find it, well, we just give up*.”

Despite all the difficulties reported above, most (17) significant others finally managed to receive help for the suicidal person or suicide attempter after several attempts.

### 3.2. Significant Others’ Perception of Patient/Client Care for the Suicidal Person/Suicide Attempter

All but one suicidal person or suicide attempter concerned by our study (one subject was not aware of any professional care received by the suicidal person or suicide attempter) were finally admitted into patient care for their suicidal or comorbid disorder by at least one professional or institution. The professionals involved were mostly psychologists/psychiatrists (in all but 2 situations) and general practitioners (one-third). At an institutional level, suicidal persons or suicide attempters had mostly been admitted in psychiatric hospitals (13 situations) and/or general hospitals (11 situations). The majority of suicidal persons or suicide attempters had been treated simultaneously or successively by several different professionals and structures.

About one-quarter of the interviewees reported positive experiences regarding the care provided to the suicidal person or suicide attempter including a feeling of safety derived from what was felt as an adequate handling of the situation by professionals, significant and helpful psychological therapeutic work provided to the suicidal person or suicide attempter, a warm reception at the psychiatric hospital both for the suicidal person or suicide attempter and their significant others, investigation of the psychological issues by general practitioners, and competence and positive communication and collaboration between professionals, such as being given factual information.

SAM: “*And in the evening, at 19h15, the psychiatrist calls me and then she tells me ‘you know, I have contacted your husband’s GP, and we have decided to give up half of another drug’. I found this fantastic!*”

However, just over three-quarters of the interviewees also expressed some dissatisfaction with the care provided to the suicidal person or suicide attempter. A recurring complaint concerned the quality of the treatment received by the suicidal person or suicide attempter; it was often considered by significant others as not having been intensive enough.

EAI: “*I was very disappointed with the care he received at the psychiatric hospital. I thought they would do a little therapy, something, but they just locked him up for three weeks and then finished, no therapy, nothing*.”

Numerous interviewees also mentioned some issues with suicide prevention, in particular that the access to means, particularly drugs, had not been restricted; a feeling that the severity of the person’s suicidality had not been properly assessed; or, further still, feeling that the intervention plan and treatment had not seemed, to the significant others, adequate in the long run in regard to the severity of the situation. Lack of follow-up after hospital discharge was frequently evoked:

SAM: “*After, there is nothing, after those 6 weeks in hospital. Then, nobody had told us he needed to see a psychiatrist so at that point we felt we had more or less been dumped*.”

Moreover, about one-quarter of interviewees regretted the lack of consistency in the care provided.

AUI: “One week, she has a female doctor, two weeks later, it’s a man...what a joke, there is no way proper care can be provided this way!”

Misinformation or poor information flow between various professionals concerning the care provided to the suicidal person or suicide attempter were frequently reported (nearly by each significant other). The significant others felt that, in order to ensure quality of care, they had to overcome this lack of proper information flow by informally endorsing the role of “case manager” or “reporter”, for example by transmitting information about the medication or treatment to psychiatrists in private practice following the suicidal person’s or suicide attempter’s discharge from hospital.

Dissatisfactions with patient care, as felt by significant others, amplified their feelings of exhaustion and hopelessness, and could sometimes generate tensions in their contacts with professionals.

### 3.3. Significant Others’ Perception of Their Collaboration with Professionals

In their search for help or when the suicidal person or suicide attempter was in patient care, especially when they acted as reference persons, significant others had to meet with health professionals and social workers. Nearly half of the interviewees reported positive experiences with professionals while the suicidal person or suicide attempter was in patient care. They felt they were considered as partners by mental health care providers because they were informed and included in the care, and they felt empathy or even received support from them.

SAM: “*Because with the psychiatrist, it goes like this: I go there with my husband, we have a little chat, and then I leave, and they talk together*.”

Professional care usually brought some relief to significant others; nevertheless contacts with professionals were sometimes difficult and could lead to dissatisfaction. More than one-third of significant others expressed regrets at not having been considered as valid representatives or partners at different times while the suicidal person or suicide attempter was under professional care. For example, they could not get information on the person’s health, particularly on the suicidal state (the reason given being that she/he was an adult and that the professional needed to abide by professional secrecy and confidentiality), on the treatment, or on important decisions taken by professionals concerning the suicidal person or suicide attempter, even when those decisions had profound impact on their relatives, such as privation of liberty, release from hospital, or if the person needed to stay at a relative’s place.

AUM: “*I was never asked ‘do you agree to take him (adult son Ed.) home this week-end?’ no, I never had a single doctor call me to ask whether I agreed or not.*”

At the same time, this mother was encouraged not to visit her son in the psychiatric hospital during the week and to take some time off such as a vacation.

Moreover, significant others often felt that professionals had very little interest in and did not take into account the information and opinions that were shared with them regarding the issues concerning the suicidal person or suicide attempter and his/her care. They felt, in particular, that their worries about the suicidality of the suicidal person or suicide attempter were sometimes not taken seriously enough by professionals or that they were being interpreted by professionals merely as difficulties experienced by the significant others him/herself.

AUM: “*It was a few months, maybe two or three months before his first attempted suicide and hospital stay that the psychiatrist told me ‘Madam, you have to put things into perspective a little bit. This young man is doing fine’. To her, I was the one with the problem*.”

Lastly, significant others did not feel that they were considered as partners by professionals as, in most cases, not only were they not given factual information about the suicidal person or suicide attempter’s situation or treatment but professionals also refused to meet with them in the absence of the suicidal person or suicide attempter. They would indeed have liked to exchange crucial information with professionals, information that could not possibly be given or requested in the presence of the suicidal person or suicide attempter, such as how to deal with firearms or other possible suicidal means in the household or information about compliance with the treatment or daily management of suicidality. The feeling of not being considered as a valid representative by the professionals in charge of the suicidal person or suicide attempter often generated anger in significant others. Similarly, not being informed, having to be very persistent in order to get information, or feeling ignored and not listened to amplified the anxiety they felt with regard to the suicidal person. These situations were particularly difficult to cope with for significant others who were very involved and giving of themselves day after day to support the suicidal person or suicide attempter; these were generally family members. Some of them began to doubt their ability to help the suicidal person or suicide attempter. A feeling of helplessness was also sometimes experienced by significant others when faced with the lack of acknowledgment of their worries, expressed on numerous occasions, about the risk of suicide. Some felt confused by messages that professionals were addressing to them, which were perceived as contradictory; on the one hand, they were told to not get involved in the care provided by professionals, to not interfere with the treatment, being sometimes seen as contributing to the suicidality; and on the other hand, they were told to take care of the suicidal person or suicide attempter on particular occasions when the professionals were not available (holidays, week-ends, nights, on release from hospital).

With regard to the support received from professionals involved in the care of the suicidal person or suicide attempter, significant others deplored what was considered as a lack of empathy demonstrated by some professionals to whom they talked about their difficulties in coping with the situation. In some situations, significant others felt that professionals were not open to acknowledge their feelings and to offer emotional support.

AUM: “*I said to the doctors ‘but me, I need help, I need help’, and I was crying and I didn’t have any tissues and nobody offered me any, and then, everybody was watching me cry, nobody said anything.*”

Interviewees would have liked those professionals to pay more attention to their experience as a close relative or significant others to a suicidal person or suicide attempter or even to offer some sort of support (theirs or that of other professionals) to cope with the situation. They reported the need to be educated in suicide matters and their expectation that the professionals would provide this sort of education.

UTT: “*Which solution? How to react? This is how I feel that relatives, they need help in these situations.*”

## 4. Discussion

Our results provide new and challenging insights about the help-seeking process, its outcomes, and the interactions that take place between significant others and professional care providers. As in previous studies [10,11,13,14,15,29], our findings show that most significant others were actively involved in help-seeking actions for the suicidal person or suicide attempter and that they endorsed an important support role or even constituted a protective and preventive factor for suicide.

Firstly, our research demonstrates that significant others were instrumental in the suicidal person or suicide attempter’s access to care even though, as in previous studies [13,15], they did not always know whom to turn to. GPs and mental healthcare professionals (mostly psychiatrists) were the professionals most sought out; they were asked to intervene but also to provide information or directions to significant others on how to better support the suicidal person or suicide attempter. Unfortunately, their help-seeking actions seemed mostly difficult, were seldom successful on the first attempt, and were filled with multiple obstacles. Indeed, it took significant others several attempts and much time and energy to finally find support and proper care for their loved one, as their requests for help were sometimes in vain. This was mostly due to the fact that professionals were not available or that in some cases, according to the significant others, they did not seem to take the suicidality seriously enough or support significant others in a concrete way [10,13]. The suicidal person or suicide attempter’s initial refusal to accept help also played a major role in about half of the situations. All these reasons created feelings of frustration and anxiety, sometimes to the point that significant others gave up seeking help. This is likely to create great risk and have dangerous consequences because a large proportion of people who complete suicide do not seek help and are not receiving any treatment by mental health specialists at their time of death [17,30]. Moreover, the deterioration, or even the disappearance of family and social support, plays an important role in the acting out of suicide [14]. Therefore, significant others should be acknowledged in their role of help-seekers and promptly supported in their efforts.

Secondly, our research shows that three-quarters of significant others were dissatisfied with the help provided by professionals to their loved ones. This was mainly caused by the difficulty to obtain rapid and truly efficient patient care or by the fact that specific care for suicidal behavior did not seem to be provided to their loved one. The scarcity of resources in the provision of care in the cantons of Fribourg and Valais, as well as a lack of services tailored specifically to suicidality management, might explain, at least partially, the reported obstacles to care access and the dissatisfaction with care. The mental health system provision is undergoing major reorganisation in these cantons, with more focus on education of professionals in suicidality issues and an improved continuity of care, especially between residential and ambulatory services. Nevertheless, continuity of care and communication obstacles seem to still present challenges to this date, according to interviews carried out recently with family members of hospitalised patients. Hopefully, the ongoing reorganisation will prevent difficult situations such as those highlighted by significant others with regard to quality of care, follow-up, and communication among professionals. Indeed, significant others sometimes felt that they, instead of the professionals in charge, were managing the case, especially when the professionals were unavailable or when communication between different professionals was lacking or difficult. Improvement in both interprofessional communication and cooperation and the continuity of care was desired by our interviewees as well as by those participating in previous studies [13]. The quality and consistency of care is also likely to foster good communication and cooperation between significant others and professionals.

Thirdly, our study demonstrates that the interaction and cooperation between significant others and professionals were still tainted to some degree by a misappreciation of the respective roles for about half of the participants, with possible misunderstandings, miscommunication, and tensions. In general, significant others did not feel sufficiently informed about or included in the care of their loved one, particularly in the case of a hospitalisation. The major issues revolved around three key elements: confidentiality, being considered as partners in the treatment, and being supported as caregivers.

The difficulties between significant others and professionals often emerged in relation to the sharing of information on the condition and the suicidal state of the suicidal person or suicide attempter. While understanding the patient’s right to confidentiality, significant others expressed their need to be aware of what was happening in order to provide appropriate support. The importance of involving significant others and giving them the means to act appropriately by sharing information has been highlighted in previous research. It has been suggested that this issue should be addressed as early as possible in the care program and a realistic and pragmatic approach adopted [13].

Participants often felt frustrated about not being considered as partners and included in the treatment by professionals. Our research highlights that the significant others of a suicidal person or suicide attempter may often be confronted with the cautious attitude of professionals who may consider them as part of the problem instead of the solution [9,13,31]. Negative life events, family discord, or demeaning behaviours have been the main reasons behind this stance [31]. However, while it is important to take these aspects into account, significant others perceive themselves as constituting the main support for and holding crucial information regarding the health and situation of the suicidal person or suicide attempter. They have “a contextualized understanding of the person experiencing a suicide crisis” [9] (p. 297). Acknowledging the information they may provide and the role they play, while respecting the patient’s rights, is likely to improve the quality of the elements on which the suicidality is assessed and on the consecutive intervention plan, especially in emergency situations. Similarly, the question of significant others’ involvement in treatment or care could be the subject of a joint reflection and decision on the roles and competencies of each party. This could lead to better patient care and synergies between different players [9,10,22,32] and be beneficial to all parties involved (patients, significant others, and professionals).

Providing information about treatment, available options, and patient care offers (professionals, structures) is also likely to create more positive interactions between significant others and professionals, to facilitate access to treatment, and to improve patient care. In fact, these actions have proved to be effective within the mental health field at large [33]. In a recent pilot study on family-centered brief intensive treatment, positive improvements for persons with acute suicide risk were found when the family was involved [34]. Tremblay [35] also advocates the involvement of the parents of young suicidal persons or suicide attempters in suicide prevention or intervention, together with the training of professionals, not only on the technical aspects of suicidal patient care but also on the collaboration and communication with a patient’s significant others.

With regard to the third kind of difficulty mentioned, and as in previous studies [10,13], this research demonstrates that significant others often felt that the realities they experienced were not acknowledged or sufficiently taken into account by professionals. They felt that they rarely received support by professionals in their struggle, whether in the form of emotional support, advice or education in suicide matters, or by being relieved or getting respite in their daily support to the suicidal person or suicide attempter. This lack of support might be due to the scarcity of resources mentioned above or to the fact that supporting significant others is not included in some professionals’ terms of reference. It would seem useful for professionals to explain their mission and the real possibilities and limitations of their intervention to significant others and to refer them to colleagues or other resources likely to support them.

Significant others should be acknowledged in their roles as care providers and supported accordingly by professionals. Responding to their requests for help would allow them to endorse their role in an appropriate and less wearing way [13], which would ultimately contribute to preventing suicidal acting out. Providing education to significant others about suicidality, suicidal crises (signs and dynamics), and crisis management may help them deal with their burden and empower them by allowing them to mobilise resources knowledgeably and with the support of professionals [9]. A suicide care education intervention improves the family caregivers’ ability to provide care and their attitudes towards the suicidal person [36]. Several interventions or approaches such as “Creativity, Optimism, Planning and Expert information” (COPE [9]) may help significant others to enhance their coping strategies [7].

In order for professionals to provide appropriate and effective care to a suicidal person or suicide attempter and to positively cooperate with their significant others in the area of suicidality intervention, they should not only be trained in suicidality screening and individual management but also receive specific training in methods and communication issues with significant others. Education in communication, such as specific interviewing with regard to suicidality [37,38], motivational interviewing in suicidal situations [39,40], or specific methods of collaboration with patients and significant others (the collaborative approach [41]; solutions-focused conversations about suicide [37]; and family caregiver empowerment in suicide prevention (COPE) [9]) are likely to provide professionals with the appropriate theoretical and clinical background and enhance their self-confidence while facing suicidality in an effective and compassionate way. Well-trained and confident professionals are more likely to meet the needs expressed by significant others in our study, as well as in previous studies [12,13,15,22,32,35]; the need to understand of the situation, to be included and acknowledged as important actors, and to receive timely and effective support [42], both for their loved ones and for themselves.

This study shows some limitations. While it is based on a rigorous research protocol and methodologically sound data, findings come from a relatively small sample of eighteen subjects within a restricted geographical and cultural area. Moreover, the suicidality in the situations discussed here is combined with recurring mental health disorders for a majority of the suicidal persons or suicide attempters, which is likely to influence the type and amount of support provided by significant others and the struggle they face.

Furthermore, data collection occurred a number of years ago. Nevertheless, the experiences of the significant others do not seem to have drastically changed since data was first collected. Indeed, seven participants contacted one of the authors on several occasions to share news, update experiences, and ask for support over a period of four years following the interview. Moreover, five interviews carried out in 2016 with the significant others of hospitalised patients showed that the issues with regard to help seeking and cooperation with professionals remain largely the same. Likewise, although the mental health facilities called upon for support at the time underwent organizational restructuration since the collection of data, they still have the same references of terms and suffer from a paucity of resources. It is also very likely that the modalities and means of recruiting (institutions, advertisements) the subjects for the study have induced biases that are difficult to measure. The recruiting was voluntary, and for seven participants it occurred through intermediary contact persons. It then becomes impossible to know how the recruitment was impacted by the role played by the intermediary contact person or whether the persons who took part in the study constitute a representative sample of significant others as a group, or if, for instance, they were more affected by their support role or more dissatisfied with the care received by the suicidal person or suicide attempter or their collaboration with professionals than other significant others who did not participate in the study.

Significant others perceived their relationship to the suicidal person or suicide attempter as globally positive, even though some tensions and conflicts arose at times; for example, when the suicidal person or suicide attempter did not want the significant others to seek help outside the family circle. A history of interpersonal conflicts or family issues may affect the readiness and persistence of significant others in seeking help.

Moreover, our sample is mostly composed of women, which is in line with the results of other studies, in which the majority of the care providers are female. Nevertheless, this could also generate a gender bias, as well as the fact that most interviewees were of middle class status. Finally, this study focuses on the point of view and perceptions of significant others. The perceptions and experiences of professionals should also be investigated and taken into account. Nevertheless, the above-mentioned biases are common and shared in studies related to suicidality.

This study has some strengths: to our knowledge, it is one of the very few studies carried out to investigate significant others’ perspectives on their experiences as the natural carers of a suicidal person or suicide attempter. It adopted a qualitative approach with a unique focus on the interactions between significant others and professionals. It focused on the significant others of adult suicidal persons or suicide attempters, which is quite rare. As such, it allowed the investigation of specific challenges of the help-seeking process and care provision, such as the confidentiality issue. Further investigations are needed in this regard, as well as research about suicide prevention education for significant others.

## 5. Conclusions

The results presented here shed new light on the practical and interactional aspects of the help-seeking process reported by significant others of a suicidal person or suicide attempter, as well as on the difficulties met along the way. They also give information on the significant others’ perception of the care received by their loved ones and the interactions experienced with the professionals who provided the care.

Significant others often felt that they had faced a series of challenges when seeking help for their suicidal loved one; these included the availability and quality of care, cooperation with professionals, especially with regard to confidentiality, inclusion in the care program, and receiving support. In light of these results, it seems appropriate to facilitate help-seeking actions by the significant others of the suicidal person or suicide attempter and to consider them as partners in the professional care provided. Moreover, effective communication and more support from professionals would allow them to assume their role as natural helpers in an appropriate manner and to reduce possible challenging interactional or relational issues, both with the suicidal person or suicide attempter and the care providers.

As for professionals, specific education on how to positively communicate and collaborate with significant others in emergency or life-threatening situations is likely to improve their feelings of competence and reduce possible conflictual or risk situations.
